# A Manuel of Organic Materia Medica

**Published:** 1900-06

**Authors:** 


A MANUEL OF ORGANIC MATERIA MEDICA.—Being a guide to materia medica of the vegetable and animal kingdoms for the use of students, druggists, pharmacists and physicians. By John M. Maisch, Ph. M., Phar. D., late Professor of Materia Medica and Botany in the Philadelphia College of Pharmacy. Seventh Edition. Revised by Henry C. Maisch, Ph. G., Ph. D., Professor of Materia Medica and Botany in the Medico-Chirurgical College of Philadelphia, Department of Pharmacy. 285 illustrations. 523 pages. Lea Brothers & Co., Philadelphia and New York. 1899.

This work has been a standard for many years. The elder Maisch made it so. His son now takes it up and revises it in its seventh edition, and fully maintains the high standard set by his father. The work has been brought up to date, including all recent advances. It harmonizes with the United States and British Pharmacopœias, and is in the same convenient size which characterized previous editions.

